# Sepsis and underlying comorbidities in intensive care unit patients: Analysis of the cause of death by different clinicians—a pilot study

**DOI:** 10.1007/s00063-023-01037-4

**Published:** 2023-06-28

**Authors:** Daniel O. Thomas-Rüddel, Holger Fröhlich, Daniel Schwarzkopf, Frank Bloos, Reimer Riessen

**Affiliations:** 1 Jena University Hospital, Center for Sepsis Control and Care, Friedrich Schiller University Jena, Jena, Germany; 2 Jena University Hospital, Department of Anesthesiology and Intensive Care Medicine, Friedrich Schiller University Jena, Jena, Germany; 3 Department of Internal Medicine, Medical Intensive Care Unit, University Hospital of Tuebingen, Tübingen, Germany; 4 Department of Internal Medicine, Klinikum am Steinenberg, Reutlingen, Germany; 5 Present Address: Department of Anesthesiology and Intensive Care Medicine, Jena University Hospital, Am Klinikum 1, 07747 Jena, Germany

**Keywords:** Sepsis, Cause of death, Comorbidity, Multimorbidity, Septic shock, Sepsis, Todesursache, Komorbidität, Multimorbidität, Septischer Schock

## Abstract

**Background:**

There is an ongoing debate as to whether death with sepsis is primarily caused by sepsis or, more often, by the underlying disease. There are no data on the influence of a researcher’s background on such an assessment. Therefore, the aim of this analysis was to assess the cause of death in sepsis and the influence of an investigator’s professional background on such an assessment.

**Materials and methods:**

We performed a retrospective observational cohort study of sepsis patients treated in the medical intensive care unit (ICU) of a tertiary care center. For deceased patients, comorbidities and severity of illness were documented. The cause of death (sepsis or comorbidities or both combined) was independently assessed by four assessors with different professional backgrounds (medical student, senior physician in the medical ICU, anesthesiological intensivist, and senior physician specialized in the predominant comorbidity).

**Results:**

In all, 78 of 235 patients died in hospital. Agreement between assessors about cause of death was low (κ 0.37, 95% confidence interval 0.29–0.44). Depending on the assessor, sepsis was the sole cause of death in 6–12% of cases, sepsis and comorbidities in 54–76%, and comorbidities alone in 18–40%.

**Conclusions:**

In a relevant proportion of patients with sepsis treated in the medical ICU, comorbidities contribute significantly to mortality, and death from sepsis without relevant comorbidities is a rare event. Designation of the cause of death in sepsis patients is highly subjective and may be influenced by the professional background of the assessor.

**Supplementary Information:**

The online version of this article (10.1007/s00063-023-01037-4) contains supplementary material, which is available to authorized users.

## Abstract (de)

**Hintergrund:**

Es wird immer wieder diskutiert, ob der Tod bei Sepsis überwiegend durch die Sepsis oder häufiger durch eine Grunderkrankung verursacht wird. Es gibt keine Daten über den Einfluss des Hintergrunds eines Forschers auf eine solche Beurteilung. Ziel dieser Analyse war es daher, die Todesursache bei Sepsis und den Einfluss des beruflichen Hintergrunds des Beurteilers auf seine diesbezügliche Einschätzung zu bewerten.

**Methodik:**

Wir führten eine retrospektive Kohortenstudie an Sepsispatienten durch, die auf der medizinischen Intensivstation eines tertiären Versorgungszentrums behandelt wurden. Bei verstorbenen Patienten wurden Komorbiditäten und der Schweregrad der Erkrankung dokumentiert. Die Todesursache (Sepsis oder Komorbiditäten oder beides zusammen) wurde unabhängig voneinander von 4 Beurteilern mit unterschiedlichem beruflichem Hintergrund (Medizinstudent, Oberarzt der medizinischen Intensivstation, anästhesiologischer Intensivmediziner und auf die vorherrschende Komorbidität spezialisierter Arzt) evaluiert.

**Ergebnisse:**

Insgesamt starben 78 von 235 Patienten im Krankenhaus. Die Übereinstimmung zwischen den Beurteilern bezüglich der Todesursache war gering (κ 0,37; 95 %-Konfidenzintervall 0,29–0,44). Je nach Beurteiler wurde in 6–12 % der Fälle die Sepsis als alleinige Todesursache festgestellt, in 54–76 % die Sepsis gemeinsam mit den Komorbiditäten und in 18–40 % nur die Komorbiditäten.

**Diskussion:**

Bei einem relevanten Anteil der auf der medizinischen Intensivstation behandelten Sepsispatienten tragen Komorbiditäten wesentlich zur Mortalität bei und der Tod durch Sepsis ohne relevante Komorbiditäten ist ein seltenes Ereignis. Die Beantwortung der Frage nach der Todesursache bei Sepsispatienten ist in hohem Maß subjektiv und kann durch den beruflichen Hintergrund des Beurteilers beeinflusst werden.

## Background

While the preventability of sepsis-associated death is still being debated [16, 21], it is mostly assumed that sepsis-associated death is caused by sepsis [24]. However, some authors challenge this assumption, claiming that in many patients, sepsis is only a mediating factor in a death that is ultimately caused by an underlying illness and therefore not preventable by sepsis treatment [11, 16, 20, 21]. Therefore, the Surviving Sepsis Campaign has stated that a key research question is “to assess the sepsis attributable mortality” [5]. There is limited empirical data on the cause of death in patients with sepsis from case reviews [11, 16] or comparison with matched cohorts without sepsis [20]. There are no data on the influence of a researcher’s background on the assessment of cause of death in sepsis. Reliable data on mortality attributable to sepsis would be important to inform epidemiology, health care policy and resource allocation, and clinical trial design [16, 20]. Therefore, the aim of this pilot study was to assess the cause of death in sepsis from chart review and the potential influence of an assessor’s professional background on such judgement.

## Methods

This is a retrospective cohort study of sepsis patients treated in the medical Intensive Care Unit (ICU) of a German university hospital. The institutional review board approved the study and waived the need for informed consent. The study was carried out in accordance with relevant guidelines and regulations.

### Study population

Patients with sepsis defined by infection-related organ dysfunction, treated in the ICU between December 2010 and May 2015 were prospectively identified as part of the “Medical Education for Sepsis Source Control and Antibiotics” (MEDUSA) quality improvement trial [1]. Inclusion and exclusion criteria for MEDUSA are presented in the supplement (Supplemental file 1). All patients who died in hospital were included in this analysis. Patients treated in the surgical/anesthesiological ICU were not included in this study.

### Data collection

Eligible patients were exported from the database of the MEDUSA trial (OpenClinica, LLC, Waltham, MA, USA). Additional information on comorbidities and their severity (Supplemental file 1) and acute severity of illness (first 24 h after sepsis onset) was documented by chart review. The cause of death was classified into three categories:

- I: sepsis as sole cause of death;
- II: sepsis and comorbidities contributing to death;
- III: comorbidities as primary cause of death accompanied by sepsis (Supplemental file 1).

Four assessors with different backgrounds independently classified the cause of death as follows: i. a trained medical student (HF), who had documented comorbidity data from the medical records; ii. an ICU senior physician (RR), board certified in internal medicine and critical care, who had overseen the management of most of the patients; iii. one of several board-certified internal medicine specialists for the patient’s leading comorbidity (UL for gastroenterology/hepatology, PS for cardiology, MM for hematology/oncology, MH for pulmonology, and FK for all other comorbidities); and iv. an external intensivist (DTR) with a background in anesthesiology and clinical sepsis research. The latter three did their classification mainly on the basis of the discharge letter, but had access to additional clinical data on request. The internal medicine specialists (iii) were treated as one rater in the analysis.

### Data analysis

Sample size was not calculated for this exploratory analysis. Baseline characteristics of patients and treatments during the ICU stay were presented using descriptive statistics. To assess interrater agreement, interrater reliability for cause of death was measured by Fleiss’ and Cohen’s kappa (κ), presented with 95% confidence intervals (CI). The association between the number of comorbidities, as measured by the Charlson Comorbidity Index (CCI), and the judgement of cause of death was tested in a one-way analysis of variance for each assessor. A *p*-value of less than or equal to 0.05 was considered statistically significant for all tests. Estimates are presented with 95% CIs. Data were analyzed using SPSS Statistics 28 (IBM, Armonk, NY, USA).

## Results

### Patient population

**Fig. 1:**
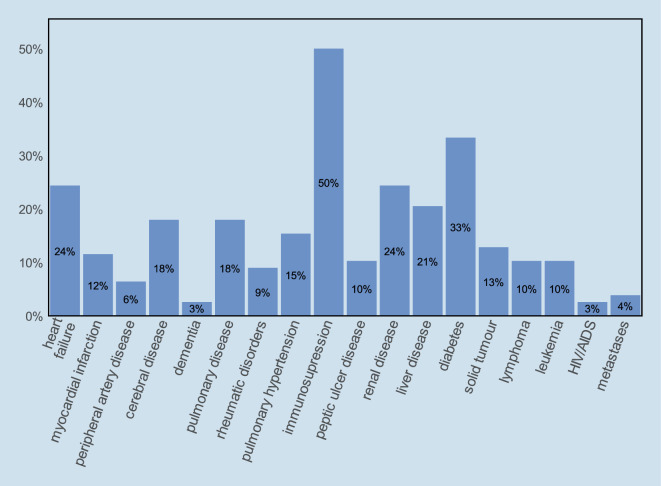
Frequency of comorbidities. *HIV/AIDS* human immunodeficiency virus/acquired immunodeficiency syndrome

During the study period, 78 (33%) of the 235 patients treated for sepsis in the medical ICU died in hospital, of whom 57 (74%) died in the ICU. Baseline clinical data and treatments are shown in Table 1. Patients had a median of 3 (IQR 2–4) comorbidities. The most frequent comorbidities were immunosuppression (*n* = 39, 50%), diabetes (*n* = 26, 33%), renal disease, and chronic heart failure (both *n* = 19, 24%; Fig. 1). The main reasons for immunosuppression were autoimmune diseases, malignancies, and a history of transplantation (Supplemental Table A1, Supplemental file 1).

**Table 1:** Patient characteristics

Characteristics	Descriptive statistics (*n* = 78)
*Age (years)*	67 [56–78]
< 50	11 (14)
50–59	12 (15)
60–69	18 (23)
70–79	25 (32)
≥ 80	12 (15)
*Sex (male)*	43 (55)
*Preadmission health (KNAUS index)* [10]	
Class A (Normal health status)	4 (5)
Class B (Moderate activity limitation)	21 (27)
Class C (Severe activity limitation due to chronic disease)	21 (27)
Class D (Bedridden patient)	32 (41)
*Number of comorbidities*	3 [2–4]
*Charlson Comorbidity Index (CCI) *[4]	3 [2–4]
*CCI with age adjustment *[3]	5 [4–7]
*Origin of infection*	
Community acquired	33 (42)
Nosocomial	45 (58)
Nursing home	4 (5)
Normal ward	37 (47)
ICU/IMC	4 (5)
*SOFA *[23]	11 [8–13]
*SAPS 2 *[12]	56 [43–69]
*APACHE 2 *[9]	28 [23–32]
*Sepsis Severity Score (SSS) *[14]	83 [74–95]
*Vasopressor [mg]*^*a*^	40 [19–95]
*CPR*	13 (17)
Out of hospital	0 (0)
IHCA, outside ICU	3 (4)
IHCA, in ICU	10 (13)
*Mechanical ventilation*	51 (65)
Duration of MV (h)^**a**^	64 [23–221]
*Renal replacement therapy*	32 (41)
Duration of RRT (h)^**a**^	45 [25–92]

Data are expressed as median [Q1–Q3] or number and percentage, *n* (%)

*CCI* Charlson Comorbidity Index, *ICU* Intensive Care Unit, *IMC* Intermediate Care Unit, *SOFA* Sequential Organ Failure Assessment Score, *SAPS II* Simplified Acute Physiology Score II, *APACHE II* Acute Physiology And Chronic Health Evaluation II, *IHCA* In-Hospital Cardiac Arrest, *MV* Mechanical Ventilation, *RRT* Renal Replacement Therapy, *ICU* intensive care unit, *CPR* cardiopulmonary resuscitation

^a^cumulative dose/time over ICU stay

### Cause of death

Overall agreement of assessors was poor, with a Fleiss’ κ of 0.37 (95%CI 0.29–0.44). Agreement was slightly better for the category I “sepsis as sole cause of death” (Fleiss’ κ 0.46, 95%CI 0.37–0.55) than for the other two categories (category II, 0.32, 95%CI 0.23–0.41; category III, 0.39, 95%CI 0.30–0.48). Individual agreement between pairs of raters ranged from 0.19 to 0.56 (Cohen’s κ, Supplemental Tables A2–A7, Supplemental file 1). It was best for the following pairs: ICU senior physician and external intensivist (Cohen’s κ 0.56, 95%CI 0.40–0.73) and ICU senior physician and medical student (0.53, 95%CI 0.36–0.71). It was worst for the pair medical specialists and external intensivist (0.19, 95%CI 0.02–0.36).

**Fig. 2:**
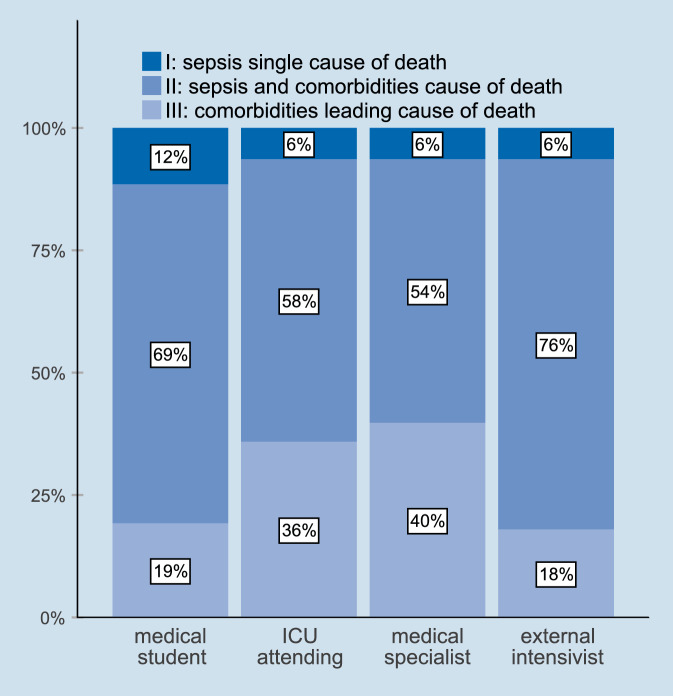
Cause of death as judged by the different assessors

**Fig. 3:**
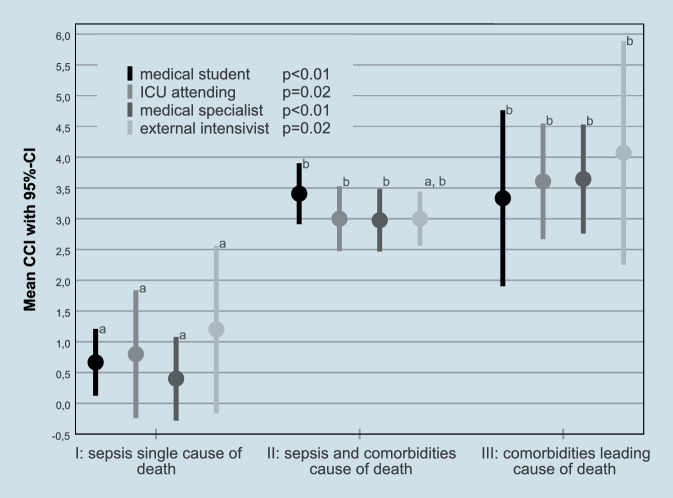
Mean Charlson Comorbidity Index (CCI) and the assessment of cause of death. *p*-values for overall difference assessed by one-way analysis of variance; *superscript letters* denote homogenous subgroups assessed by post hoc testing, *95%CI* 95% confidence interval

Depending on the assessor, sepsis was the sole cause of death in 6–12% of cases, sepsis and comorbidities in 54–76%, and comorbidities alone in 18–40% (Fig. 2). Compared with patients assessed as having died from sepsis alone, patients assessed as having died from comorbidities alone or from sepsis and comorbidities together had a higher CCI (Fig. 3). The most frequent severe comorbidities in patients classified as having died from comorbidities alone were liver disease and hemato-oncological diseases (Supplemental Figure A1, Supplemental file 1).

## Discussion

In a review of 78 deaths with sepsis treated in a medical ICU, we found that a relevant proportion of mortality was due to comorbidities. However, there was poor interrater agreement in assessing whether death in a patient with sepsis was caused by sepsis alone, by the underlying comorbidity, or by both.

When it comes to the causes of death in patients with sepsis, there is limited evidence to compare our data to. In a Welsh study focusing on patients with sepsis treated on normal wards, only 24% of deaths were considered at least possibly due to sepsis [11]. Most of the patients were frail or had limitations of therapy and therefore did not receive ICU care, making a comparison with our cohort difficult. No assessment of the objectivity of the assessment was reported in this study [11]. A Brazilian study looking at death records reclassified 80% of all cases initially documented as sepsis mortality into another underlying cause, but the exact methodology and definitions of causality are not easy to understand in the manuscript [19].

A multicenter study in the USA examined 300 deaths in which sepsis was present during hospitalization. The immediate and underlying causes of death and its potential preventability were assessed [16]. Sepsis was found to be the immediate cause of death in 198 of these cases. However, 40% of patients had end-stage comorbidities qualifying them for hospice care (mostly cancer) and 22% had limitations in their therapy on admission. The authors of the study also concluded that most underlying causes of death were related to severe chronic comorbidities. Only 36 cases were judged to be at least possibly preventable with better hospital-based care. The first 30 cases in each center were assessed by a pair of reviewers with an agreement ranging from 0.32–0.93 for cause of death and 0.34–0.60 for preventability. After retraining, agreement increased but remained below 0.7 for preventability. No detailed information on the reviewers’ background is given. The mix of ICU and normal ward cases, the different distribution of comorbidities and the different methodology limit comparability with our study. The one-third of patients in whom sepsis was not the immediate cause of death is comparable in magnitude to the 18–40% in our study who died from comorbidities. It has also been shown in a general hospital population that the vast majority of hospital deaths were due to underlying diseases [18]. Age, active cancer, and existing do not resuscitate (DNR) orders have also been described as major risk factors for mortality in sepsis patients treated in the emergency department [7].

One study assessed the attributable fraction of deaths from sepsis by comparing sepsis patients in the ICU with matched critically ill patients without sepsis and general population controls [20]. Their estimate of the attributable proportion of deaths ranged from 15 to 93%. While this method is independent of individual case assessment and assessor bias, it is highly dependent on the control cohorts and matching algorithms. To our knowledge there is no study cross-validating both approaches in the same cohort.

Autopsy studies are often considered the gold standard for determining cause of death. In the coronavirus disease 2019 (COVID-19) pandemic, they were extremely helpful in establishing causality and pathophysiology in COVID-19 deaths [8, 25, 26]. In (suspected) sepsis they have been helpful in confirming or excluding the presence of infection [2], in showing uncontrolled foci of infection [22], and in some cases in showing missed diagnoses other than sepsis [6]. However, findings in multiorgan dysfunction syndrome are often on-specific and modified by treatment or withdrawal of treatment [13]. There is often a poor correlation between tissue findings and the degree of organ dysfunction [22]. Therefore autopsy in sepsis may be helpful in determining the immediate cause of death and reasons for treatment failure, but less so in determining the association of underlying diseases and death. Due to the low autopsy rate in Germany, autopsy results were not used in our study.

Our interrater agreement is relatively low, but in the same range as in the American study before retraining. Interrater agreement has also been found to be low for other sepsis-related topics such as the presence of sepsis itself, time zero or bundle compliance [15, 17]. The highest agreement was seen for the ICU senior physician and the external intensivist, both from a critical care background, and for the ICU senior physician and the medical student who planned the study together. The lowest level of agreement was seen between the medical specialists, trained in internal medicine with limited critical care experience, and the external intensivist trained in anesthesiology and sepsis research. Including a medical student in the process could be seen as a source of bias. However, the pairwise interrater agreement between the medical student and the three other raters was no worse than that between the experienced clinicians. Reporting the number, background, training, and interrater agreement seems to be important in any further research on the subject of sepsis and cause of death.

A strength of our study is the contribution of assessors from different backgrounds, but it has several limitations. One is the single-center and retrospective nature and an assessment based on discharge letters for two of the four assessors. As only one assessor from each background participated, we cannot distinguish between the influences of professional background and personal subjectivity. A qualitative analysis of the thought processes leading to the judgements of the assessors was beyond the scope of the study. The focus on medical patients in a university hospital and the lack of surgical patients may have resulted in a higher than average burden of comorbidities and especially immunosuppression. The category “sepsis and comorbidities contributing to death” is quite broad but our initial approach to further differentiation failed due to very poor discrimination within this group by our assessors. It should therefore be seen as a pilot study to stimulate future research on this topic.

## Conclusion

In a relevant proportion of sepsis-patients treated in the medical intensive care unit (ICU), comorbidities contribute substantially to mortality, and death from sepsis without relevant comorbidities appears to be a relatively rare event. Answering the question of cause of death in sepsis patients is limited by the perspective-dependent nature of any judgement and will only be reliable once a theoretical and methodological solution to this problem has been found.

### Supplementary Information

Additional methods and results

## Declarations

The study was approved by the ethics committee of the medical faculty of the Eberhard-Karls-University Tubingen (104/2015BO2, 20 April 2015, original study title “Einfluss von Komorbiditäten auf die Mortalität bei internistischen Intensivpatienten mit schwerer Sepsis und septischem Schock”). Due to the observational nature of the study the need for informed consent was waived by the ethics committee and the data security officer of the university hospital. The multicenter study (MEDUSA) whose database was partially used was approved locally by the ethics committee of the medical faculty of the Eberhard-Karls-University Tubingen (556/2010BO2, 13 December 2010, original study title “Medical Education for Sepsis Source Control and Antibiotics (MEDUSA)”) and overall by the ethics committee of the university hospital Jena (2910–08/10, 30 November 2010, original study title “Medical Education for Sepsis Source Control and Antibiotics (MEDUSA)”) and it was registered at ClinicalTrials.gov (NCT01187134). The need for informed consent for the MEDUSA trial was waived by all involved institutional review boards and all responsible state data protection boards.
