# Supplementary material for: Sepsis and underlying comorbidities in intensive care unit patients: Analysis of the cause of death by different clinicians—a pilot study
Source: Med Klin Intensivmed Notfmed. 2023 Jun 28;119(2):123–8. doi: 10.1007/s00063-023-01037-4 (PMC10901974; doi:10.1007/s00063-023-01037-4)
Supplement: Supplementary file 1 — Additional methods and results [file 63_2023_1037_MOESM1_ESM.pdf]

Additional file 1

Inclusion and exclusion criteria

Inclusion

- proven or suspected infection
- acute onset of infection-related organ dysfunction

Exclusion

- no full commitment to therapy at onset of sepsis
- not treated on a participating ICU after sepsis diagnosis
- sepsis therapy was started in another hospital

Cause of death - Category definitions

Originally cause of death was classified using four categories. As the two middle showed very poor discrimination, they were merged for further analysis resulting in the following categories:

Category I sepsis single cause of death

The patient had died of sepsis or its direct consequences without relevant comorbidities.

Category II sepsis and comorbidities cause of death

The patient suffered from pre-existing relevant or severe comorbidities and died in association with sepsis. Both contributed to death.

Category III comorbidities leading cause of death accompanied by sepsis

Sepsis was only a secondary diagnosis or was already under control or cured. The patient died from severe comorbidities (e.g. malignancy, liver cirrhosis, COPD) independently from sepsis. These were also the main reason for limitations of therapy.

The original four categories were definded as follows:

Previous category I sepsis single cause of death

The patient had died of sepsis or its direct consequences without relevant comorbidities.

Previous category II sepsis leading cause of death but significant comorbidity

The patient had pre-existing relevant or severe comorbidities. The uncontrolled sepsis and the associated organ failure, however, was the leading cause of death. The comorbidities were not limiting prognosis.

Previous category III sepsis and comorbidities in equal measure cause of death

The patient suffered from pre-existing relevant or severe comorbidities and died in association with sepsis. However, the comorbidities were already significantly limiting the prognosis and might have prevented a successful sepsis therapy. Therefore comorbidities and sepsis can be regarded as the cause of death in roughly equal proportions. The extent of the comorbidities may also have had an impact on decisions regarding limitations of therapy.

Previous category IV comorbidities leading cause of death accompanied by sepsis

Sepsis was only a secondary diagnosis or was already under control or cured. The patient died from severe comorbidities (e.g. malignancy, liver cirrhosis, COPD) independently from sepsis. These were also the main reason for limitations of therapy.

Definition of severe comorbidity

- comorbidity was the reason for current inpatient admission.

OR

- Heart failure: NYHA IV; ejection fraction (EF) <30%
- Peripheral artery disease: Fontaine IIb, III, IV, existing gangrene.
- Cerebral disease: Severe functional limitations possibly with secondary complication (e.g. aspiration pneumonia, cerebral seizure, etc.)
- Dementia: inpatient treatment due to complications of dementia
- Pulmonary disease: GOLD IIII, O2- continuous therapy
- Immunosuppression: existing aplasia, cytostatic therapy (also outpatient)
- Pulmonary hypertension: WHO stage IV, PAP >55 mmHg with reduced RV-function
- Liver disease: Child C; MELD- score 31-40, presence of esophageal or fundus varices
- Diabetes mellitus: acute ketoacidosis, acute hyperosmolar coma, acute hypoglycemia
- Solid tumor: inpatient treatment due to complication in the course of tumor activity
- Lymphoma: inpatient treatment due to complication in the course of tumor activity (tumor lysis syndrome, etc.)
- Leukemia: inpatient treatment due to complications after completed treatment (e.g.: GvHD in case of SCX)
- HIV/AIDS: existing AIDS disease
- Metastatic tumor: presence of metastases

Additional tables

Table A1: Reasons for Immunosuppression

Reason for Immunosuppression	Frequency
lung disease	3 (8)
autoimmune disease	11 (28)
solid tumour	2 (5)
lymphoma	3 (8)
leukemia	6 (15)
liver transplant	4 (10)
renal transplant	1 (3)
bone marrow transplant	4 (10)
other	5 (13)

Data are expressed as n (%); percentages based on the 39 immunosuppressed patients

Table A2: Agreement / Disagreement of the medical student and the ICU attending regarding cause of death

Weighted Kappa: 0.53 (0.36-0.71 CI)		ICU attending		
		I	II	III
medical student	I: sepsis single cause	**5**	*3*	*1*
	II: sepsis and comorbidities	*0*	**40**	*14*
	III: comorbidities leading	*0*	*2*	**13**

Table A3: Agreement / Disagreement of the medical student and the medical specialist regarding cause of death

Weighted Kappa: 0.36 (0.18-0.54 CI)		medical specialist		
		I	II	III
medical student	I: sepsis single cause	**4**	*4*	*1*
	II: sepsis and comorbidities	*1*	**34**	*19*
	III: comorbidities leading	0	*4*	**11**

Table A4: Agreement / Disagreement of the medical student and the external intensivist regarding cause of death

Weighted Kappa: 0.42 (0.22-0.62 CI)		external intensivist		
		I	II	III
medical student	I: sepsis single cause	**3**	*6*	*0*
	II: sepsis and comorbidities	*2*	**46**	*6*
	III: comorbidities leading	*0*	*7*	**8**

Table A5: Agreement / Disagreement of the ICU attending and the medical specialist regarding cause of death

Weighted Kappa: 0.41 (0.23-0.60 CI)		medical specialist		
		I	II	III
ICU attending	I: sepsis single cause	**2**	*3*	*0*
	II: sepsis and comorbidities	*3*	**30**	*12*
	III: comorbidities leading	*0*	*9*	**19**

Table A6: Agreement / Disagreement of the ICU attending and the external intensivist regarding cause of death

Weighted Kappa: 0.56 (0.40-0.73 CI)		external intensivist		
		I	II	III
ICU attending	I: sepsis single cause	**3**	*2*	*0*
	II: sepsis and comorbidities	*2*	**43**	*0*
	III: comorbidities leading	*0*	*14*	**14**

Table A7: Agreement / Disagreement of the medical specialist and the external intensivist regarding cause of death

Weighted Kappa: 0.19 (0.02-0.36 CI)		external intensivist		
		I	II	IIII
medical specialist	I: sepsis single cause	**1**	*4*	*0*
	II: sepsis and comorbidities	*3*	**34**	*5*
	III: comorbidities leading	*1*	*21*	**9**

Additional figures

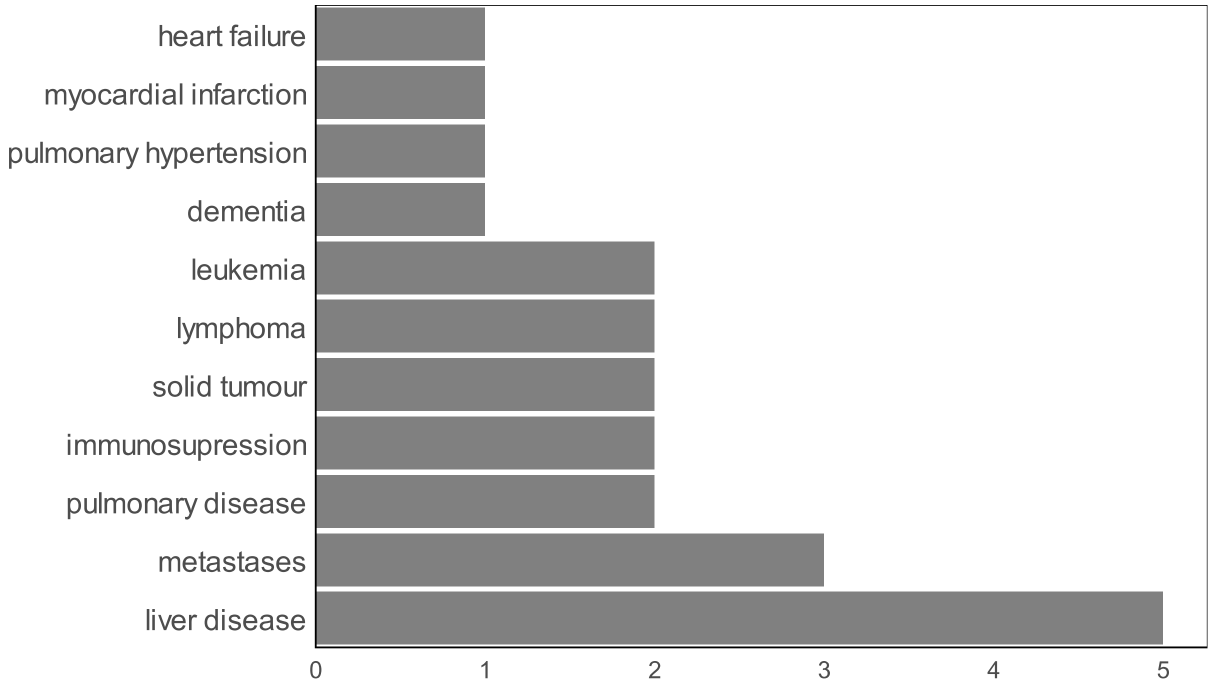

Figure A1: Frequency of severe comorbidities (definitions above) in patients assessed by at least two assessors to have comorbidities as leading cause of death

Affiliations of the medical specialist assessors listed in the acknowledgement:

Prof. Dr. Ulrich Lauer
Department of Internal Medicine VIII
University Hospital of Tuebingen
Tübingen, Germany
ulrich.lauer@med.uni-tuebingen.de

Prof. Dr. Peter Seizer
Innere Medizin I (Kardiologie)
Ostalb Klinikum Aalen
Aalen, Germany
peter.seizer@kliniken-ostalb.de

PD Dr. Dr. Martin Müller
Klinik für Hämatologie, Onkologie und Immunologie
KRH Klinikum Siloah
Hannover, Germany
martin.mueller@krh.eu

Dr. Maik Häntschel
Department of Internal Medicine VIII
University Hospital of Tuebingen
Tübingen, Germany
maik.haentschel@med.uni-tuebingen.de

Dr. Florian Kreth
Division of Emergency Medicine
Department of Internal Medicine I
University Hospital of Tuebingen
Tübingen, Germany
florian.kreth@med.uni-tuebingen.de
